# Shaping a research agenda to ensure a successful European health technology assessment: insights generated during the inaugural convention of the European access academy

**DOI:** 10.1186/s13561-022-00402-x

**Published:** 2022-11-05

**Authors:** Elaine Julian, Mira Pavlovic, Oriol Sola-Morales, Fabrizio Gianfrate, Mondher Toumi, Heiner C. Bucher, Christian Dierks, Wolfgang Greiner, Peter Mol, Jean-François Bergmann, Tomas Salmonson, Ansgar Hebborn, Mathilde Grande, Antonella Cardone, Jörg Ruof

**Affiliations:** 1 R-Connect Ltd, Basel, Switzerland; 2 Medicines Development and Training (MDT) Services, Paris, France; 3 Fundació HiTT, Barcelona, Spain; 4 University of Ferrara, Ferrara, Italy; 5 Faculty of Medicine, Public Health Department, Aix-Marseille University, Marseille, France; 6 Basel Institute for Clinical Epidemiology and Biostatistics (CEB), University Hospital Basel and University of Basel, Basel, Switzerland; 7 Dierks+Company Rechtsanwaltsgesellschaft mbH, Berlin, Germany; 8 School of Public Health, Bielefeld University, Bielefeld, Germany; 9 Department of Clinical Pharmacy and Pharmacology, University Medical Center Groningen, University of Groningen, Groningen, The Netherlands; 10 University Paris-Cité and AP-HP, Paris, France; 11 Consilium, Uppsala, Sweden; 12 Efpia, Brussels, Belgium; 13 AMEDICONSEIL, Brive-La-Gaillarde, France; 14 Cancer Patients Europe, Brussels, Belgium; 15 Medical School of Hanover, Hanover, Germany

**Keywords:** EU HTA, Uncertainty, Comparators, Endpoints, Process, Clinical Trial Design, Patient-relevance, Access

## Abstract

**Objectives:**

Key challenges for a joint European Health Technology Assessment (HTA) include consolidated approaches towards the choice of adequate comparator(s), selection of endpoints that are relevant to patients with a given disease, dealing with remaining uncertainties as well as transparent and consistent management of related processes. We aimed to further crystallize related core domains within these four areas that warrant further research and scrutiny.

**Methods:**

Building on the outcomes of a previously conducted questionnaire survey, four key areas, processes, uncertainty, comparator choice and endpoint selection, were identified. At the inaugural convention of the European Access Academy dedicated working groups were established defining and prioritizing core domains for each of the four areas. The working groups consisted of ~ 10 participants each, representing all relevant stakeholder groups (patients/ clinicians/ regulators/ HTA & payers/ academia/ industry). Story books identifying the work assignments were shared in advance. Two leads and one note taker per working group facilitated the process. All rankings were conducted on an ordinal Likert Response Scale scoring from 1 (low priority) to 7 (high priority).

**Results:**

Identified key domains include for *processes*: i) address (resource-) challenge of multiple PICOs (Patient/ Intervention/ Comparator/ Outcomes), ii) time and capacity challenges, iii) integrating all involved stakeholders, iv) conflicts and aligning between different multi-national stakeholders, v) interaction with health technology developer; for *uncertainty*: i) early and inclusive collaboration, ii) agreement on feasibility of RCT and acceptance of uncertainty, iii) alignment on closing evidence gaps, iv) capacity gaps; for *comparator choice*: i) criteria for the choice of comparator in an increasingly fragmented treatment landscape, ii) reasonable number of comparators in PICOs, iii) shape Early Advice so that comparator fulfils both regulatory and HTA needs, iv) acceptability of Indirect Treatment Comparisons (ITC), v) ensure broad stakeholder involvement in comparator selection; for *endpoint selection*: i) approaching new endpoints; ii) patient preferences on endpoints; iii) position of HTA and other stakeholders; iv) long-term generation and secondary use of data; v) endpoint challenges in RCTs.

**Conclusions:**

The implementation of a joint European HTA assessment is a unique opportunity for a stronger European Health Union. We identified 19 domains related to the four key areas, processes, uncertainty, comparator choice and endpoint selection that urgently need to be addressed for this regulation to become a success.

## Introduction

In December 2021 the European Regulation on Health Technology Assessment (HTA), a key pillar of the EU Pharmaceutical Strategy, was adopted by the Council and the European Parliament. Since January 2022, preparatory work has commenced including the stepwise setting up of a secretariat, a member states’ coordination group as well as respective subgroups, a stakeholders’ network, drafting implementing and delegated acts, and drafting guidance documents. The preparatory phase ends in December 2024 with a subsequent implementation phase running until January 2030. During the preparatory phase a limited number of Joint Scientific Consultations (JSC) will be offered and Joint Clinical Assessments (JCA) will be conducted in a step-wise approach. From January 2025 on, all cancer medicines and Advanced Therapy Medicinal Products (ATMPs) will be assessed according to these joint actions, and orphan medicines will follow from Jan 2028 onwards [1].

For the preparatory phase, a service contract was signed with the EUnetHTA 21 joint consortium that is led by the Dutch ‘Zorginstituut’ (ZIN) and includes a total of 13 European HTA bodies. The service contract includes a wide variety of activities building on the achievements and lessons learned from the EUnetHTA Joint Actions and supporting the stepwise implementation of the EU HTA regulation [2]. The EUnetHTA 21 work agenda covers a various deliverables including e.g., the development of methodological and process guidances and the conduct of a limited number of JSCs and JCAs until 2025 [3].

**Fig. 1:**
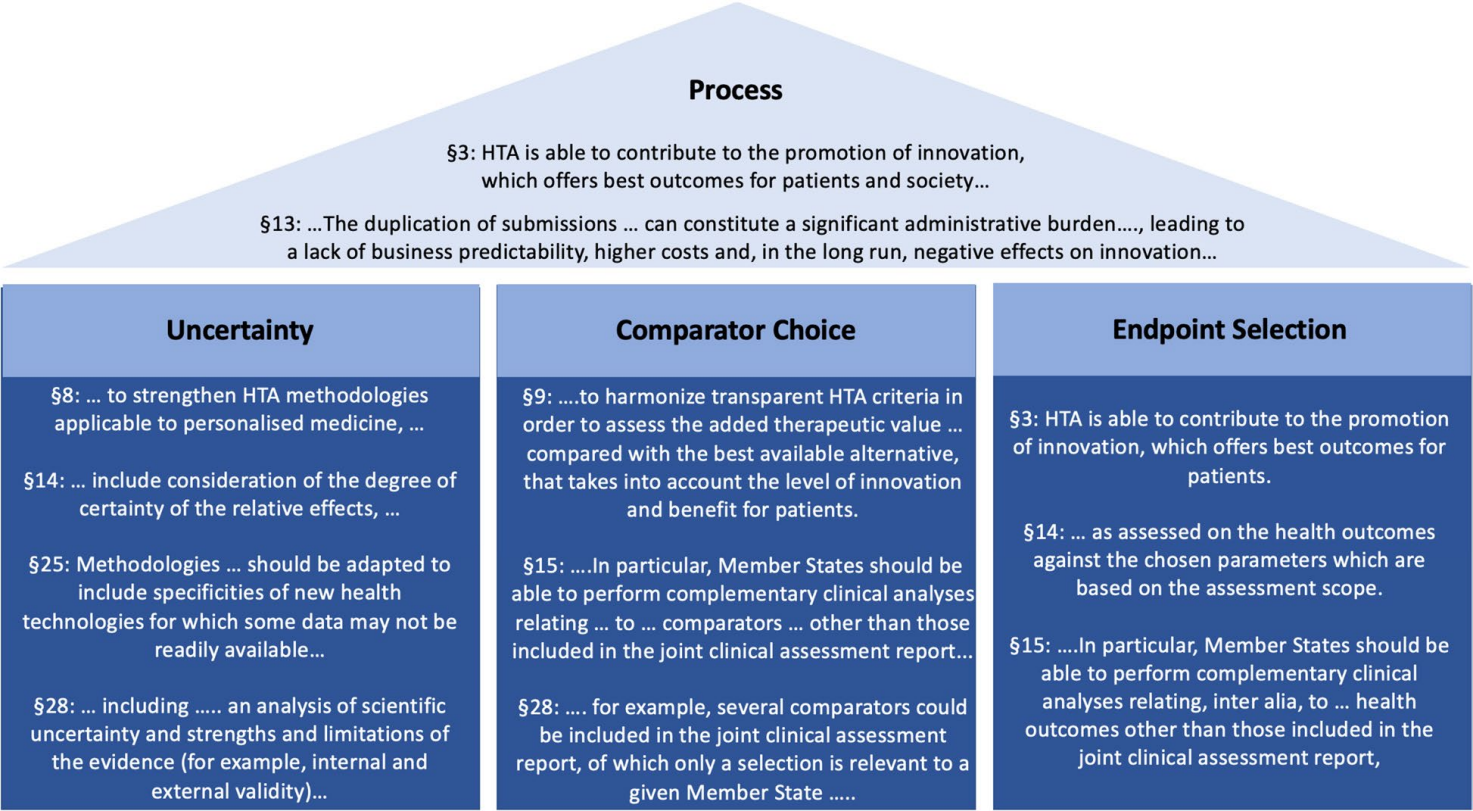
EU HTA Regulation language related to the identified challenge areas. Language derived from the preamble of the EU HTA Regulation regarding the four key areas that need to be addressed in order for the EU HTA Regulation to provide an ‘additional benefit’ compared to the status quo of many parallel independent national and subnational assessments

Parallel to those publicly funded activities to implement the EU HTA regulation, the ‘European Access Academy’ (EAA) was founded as a self-organized, crowd funded initiative aiming to facilitate and further support the shaping of a joint European Value Framework in order to meet the regulation’s vision *‘…to address unmet medical needs and facilitate access to innovative medicines …’* [4]. Specifically, the regulation includes extensive language guiding its implementation (Fig. 1) suggested to ensure that this regulation will strengthen the European Health Union. During the inaugural convention of the EAA a research agenda was developed highlighting key challenges areas for a joint European HTA and crystallizing related domains that warrant further research and scrutiny.

## Methods

**Fig. 2:**
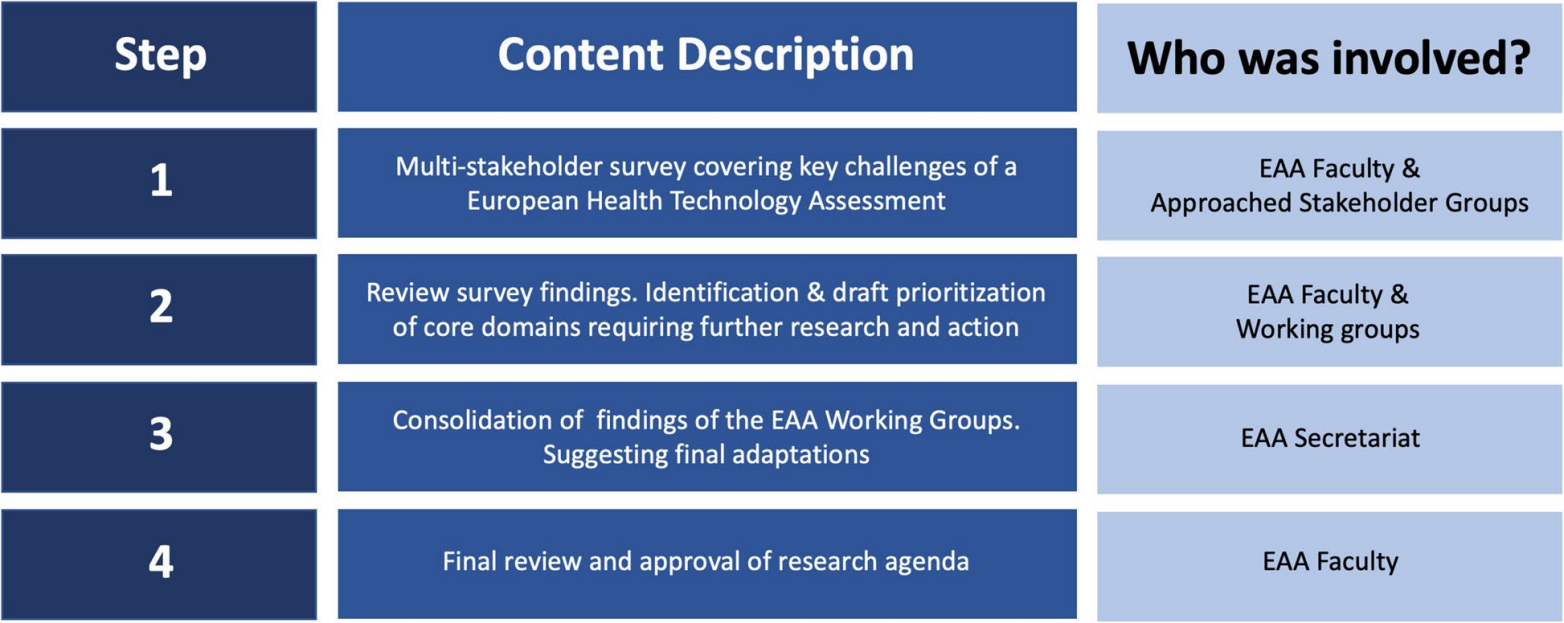
The four steps in the development of the EAA’s research agenda

A total of four procedural steps were applied to determine a research agenda that focuses on achieving an additional benefit of a joint European HTA assessment over the existing national procedures. The four steps comprise i) a preparatory multi-stakeholder survey; ii) draft identification and prioritization of key domains in a working group format; iii) consolidation of the findings of the working groups; iv) final review and approval of the research agenda. An overview of the four steps is displayed in Fig. 2.

- Step 1: Multi-stakeholder Survey:

Prior to the inaugural convention of the EAA a multi-stakeholder survey was conducted. The semiquantitative questionnaire was developed leveraging a modified Delphi procedure and circulated across a total of *n* = 189 European stakeholder institutions including HTA and regulatory bodies, clinical oncology associations, patient representatives, and industry associations. Respective findings from the *n* = 30 responses (HTA bodies: 9; regulators: 10; patients’ and physicians’ associations: 3 each; industry: 5) were analysed and grouped into the four key challenge areas: i) processes, ii) uncertainty, iii) comparator choice, and iv) selection of endpoints that are relevant to patients. A project report was shared with the EAA faculty prior to the inaugural convention and submitted for publication [5].

- Step 2: Draft Identification and Prioritization of Key Domains:

The inaugural convention of the European Access Academy was attended by 26 participants on-site and an additional 142 Unique Viewers via ZOOM during the Public Session. The EAA Working Session was set up as a hybrid meeting, allowing participation both on-site and remotely via ZOOM, and had a total of 37 participants. Building on the outcomes of the survey, four dedicated working groups were established identifying, defining, and prioritizing key domains related to the four above mentioned challenge areas. The working groups consisted of 8—10 participants each, representing a variety of involved stakeholder groups (patients/ clinicians/ regulators/ HTA & payers/ academia/ industry). The questionnaire findings as well as story books outlining the work assignments for each of the working groups were shared in advance. Two leads representing the EAA faculty and one note taker per working group facilitated the process. After the conceptual work of identifying, defining and prioritizing key domains within the working teams, results were shared across all EAA workgroups, and all participants were asked to rank relevance of the related domains from their specific point of view. Pre-generated QR codes were shared to allow for simultaneous IT based ranking using online forms generated with Microsoft Office Online. All rankings were conducted on an ordinal Likert Response Scale scoring from 1 (low priority) to 7 (high priority).

- Step 3: Consolidation of the Findings of the Working Groups

**Fig. 3:**
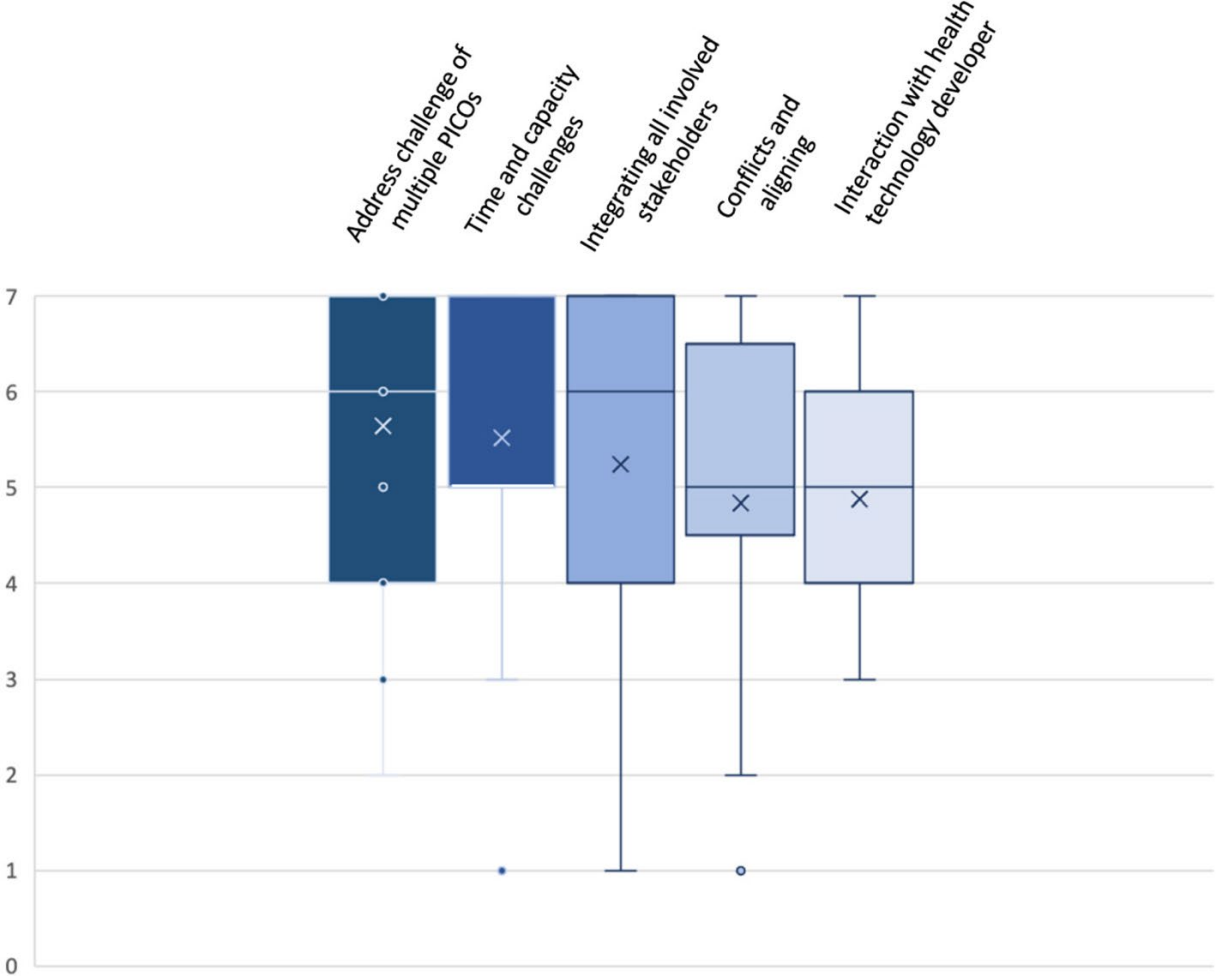
Box Plot: Ranking of Key Domains related to Challenges with Processes. Indicated are mean (x); median (bar in coloured area); interquartile range (coloured area), any individual ranks that were chosen (dots); and min/ max whiskers (dots lying outside of the whiskers are considered outliers); all rankings were conducted on an ordinal Likert Response Scale scoring from 1 (low priority) to 7 (high priority)

**Fig. 4:**
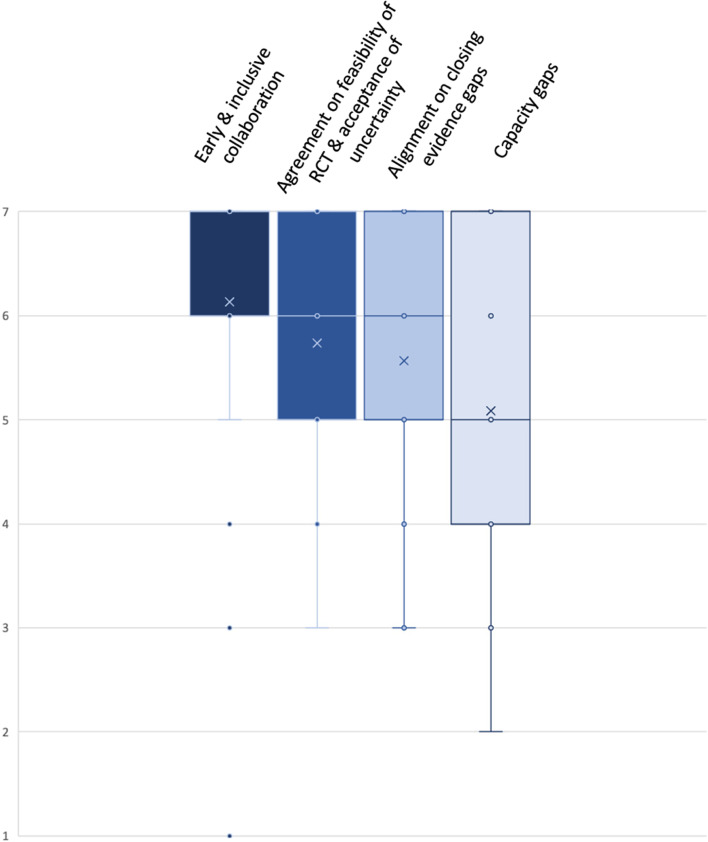
Box Plot: Ranking of Key Domains related to Challenges with Uncertainty. Indicated are mean (x); median (bar in coloured area); interquartile range (coloured area), any individual ranks that were chosen (dots); and min/ max whiskers (dots lying outside of the whiskers are considered outliers); all rankings were conducted on an ordinal Likert Response Scale scoring from 1 (low priority) to 7 (high priority)

**Fig. 5:**
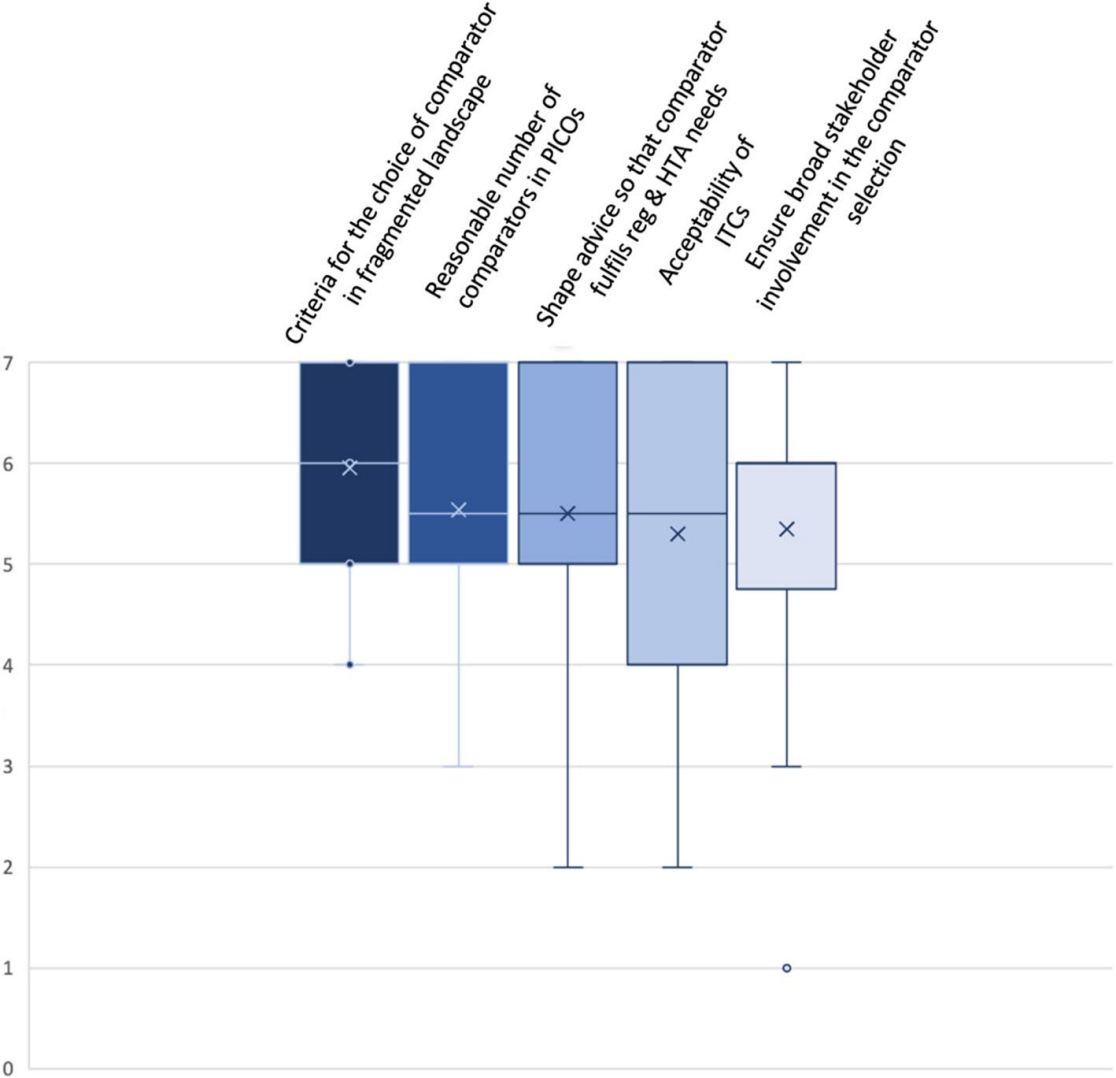
Box Plot: Ranking of Key Domains related to Challenges with Comparator Choice. Indicated are mean (x); median (bar in coloured area); interquartile range (coloured area), any individual ranks that were chosen (dots); and min/ max whiskers (dots lying outside of the whiskers are considered outliers); all rankings were conducted on an ordinal Likert Response Scale scoring from 1 (low priority) to 7 (high priority)

**Fig. 6:**
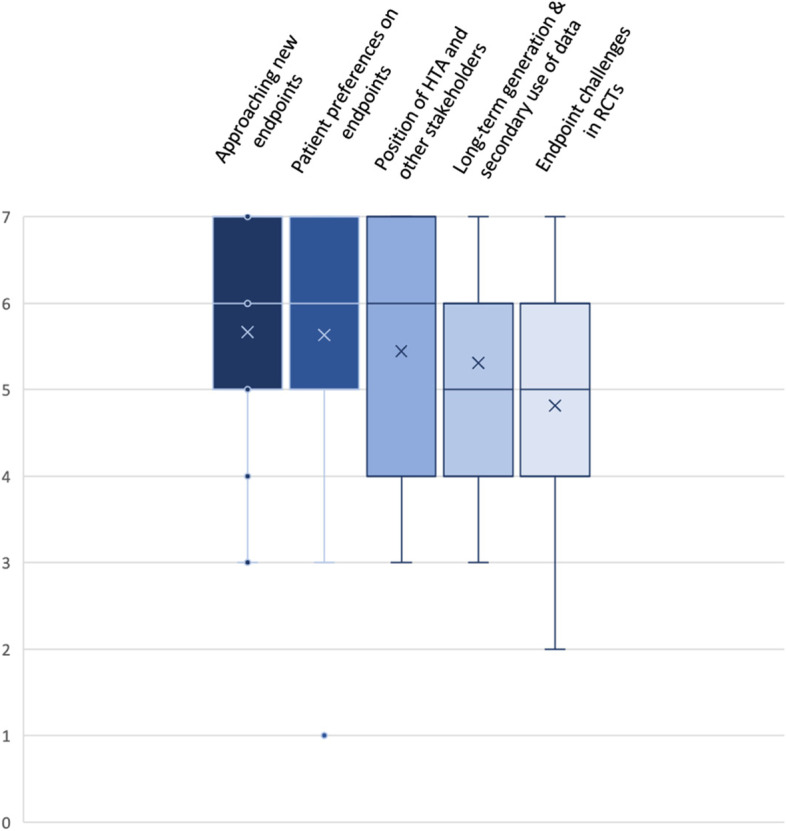
Box Plot: Ranking of Key Domains related to Challenges with Endpoint Selection. Indicated are mean (x); median (bar in coloured area); interquartile range (coloured area), any individual ranks that were chosen (dots); and min/ max whiskers (dots lying outside of the whiskers are considered outliers); all rankings were conducted on an ordinal Likert Response Scale scoring from 1 (low priority) to 7 (high priority)

In a next step findings and rankings of the EAA working groups were reviewed by the EAA secretariat (EJ, JR). Descriptive statistics were applied to the rankings derived from the EAA convention including graphical display as Box Plots (see Figs. 3, 4, 5 and 6). All analyses were conducted with Microsoft Excel Version 2019. Furthermore, a content review of convention outcomes was conducted. The descriptions of each of the work domains and related guiding questions were extracted from the workgroup notes and transferred into table format (see Tables 1, 2, 3 and 4). Any duplications were removed, and various adjustments of wording were suggested to improve clarity of the recommended key domains and related questions.

- Step 4: Final Review and approval of Research Agenda

Two rounds of reviews were conducted to obtain full feed-back from the EAA faculty. In a follow up virtual meeting ~ 10 days after the EAA convention key findings and adjustments were agreed upon. Subsequently the EAA secretariat (EJ, JR) drafted the key components of the publication which was again circulated across all EAA faculty members for further review and input.

## Results

A total of 19 domains warranting further research were identified, with four domains related to uncertainty, and five each to comparator choice, endpoint, and processes.

- Key Domains related to Challenges with Processes:

A description of each of the domains and listing of guiding questions are displayed in Table 1. Descriptive statistics of the ranking (*n* = 25 responses) are presented in Fig. 3.

**Table 1:** Identified key domains to address the challenge of processes

Process Challenges					
Key Domains	Address challenge of multiple PICOs	Time and capacity challenges	Integrating all involved stakeholders	Conflicts and aligning	Interaction with health technology developer
Description	PICO is the basis for both the advice as well as the assessment. Early discussion of PICO and agreement across regulatory and HTA stakeholders is critical to design a clinical development program	Ensure sufficient capacities to allow for early and inclusive collaboration e.g., sufficient joint early advice opportunities and to allow sufficient time for submission, generation and publication of the assessment report so national processes are not impacted	EU HTA will shape future oncology care across Europe. Early and inclusive involvement of all stakeholders (patients, HTA bodies, regulators, clinicians, industry) is key to ensure success of the regulation	Overcoming current multiplicity and/or duplication of national HTA procedures is at the heart of the EU HTA regulation. Resolution of divergences between national and EU HTA bodies and between the various other stakeholders is key	Early and inclusive collaboration between clinicians, patients, regulators, HTA bodies and the industry to ensure that the developed medicines are addressing an unmet medical need
Guiding Questions	What is the rationale for different PICOs? Are there options to decrease fragmentation of PICOs? How can the challenge of a PICO changing from advice to assessment be addressed?	What is the expected number of new oncology drug applications / year and the related joint scientific advice capabilities of EMA/ EU HTA? What can be done to increase the number of available joint scientific advice slots?	How are the various stakeholders involved throughout a medicine’s HTA process? How will their input be used and where can it help improve the quality and usability of the assessment? How to ensure a strong clinical input?	How to ensure national HTA bodies incorporate the JCA into their process, improving the quality of decision-making and enabling timely patient access? How to resolve or contextualise different recommendations of regulators vs HTA bodies?	What are benefits of early and inclusive collaboration with the HTD in delivering the objectives of the Regulation? At what stages and frequency should this engagement occur?

Identified core domains include i) address (resource-) challenge of multiple PICO (Patient/ Intervention/ Comparator/ Outcomes) schemes (mean 5.6; median 6; interquartile range (IQR) 4–7), ii) time and capacity challenges (mean 5.5; median 5; IQR 5–7), iii) integrating all involved stakeholders (mean 5.2; median 6; IQR 4–7), iv) conflicts and aligning between different multi-national stakeholders (mean 4.8; median 5; IQR 4.5–6.5), v) interactions with health technology developers (HTDs) (mean 4.9; median 5; IQR 4–6).

Process challenges were repeatedly discussed within all working groups as most of the methodological challenges include process ramifications. The challenge of multiple PICOs (identified in the scoping phase of a joint assessment), i.e., the time and resources required for manufacturers to prepare the required data and ensure availability of data for each requested PICO and the time and resources required on the side of EU HTA authorities to assess data for each PICO, was e.g., also covered in the comparator working group and was considered one of the most important hurdles for the EU HTA regulation to become a success story for Europe. Time and capacity gaps were discussed in all working groups as a major issue seriously limiting the potential of the EU HTA regulation and the ability to deliver a timely, high-quality assessment. The issue of capacity is also related to the challenge of multiple PICOs, as the higher the number of PICOs is, the more resources are needed for the assessment. Integration of all stakeholder groups in the process was considered crucial to prevent the new regulation just resulting in sophisticated technical discussions between HTA bodies and HTDs. Instead, involvement of medical societies and elaboration of relevant guidelines are considered key when e.g., determining comparative treatment regimens for a given group of patients. It was repeatedly questioned whether the outcome of the EUnetHTA 21 stakeholder network’ deliverables will allow for appropriate involvement of all relevant stakeholders, representing the EU as a whole, rather than only a few dominating countries. Consequently, ‘conflicts and aligning’ was considered a highly relevant additional domain. The evolving EU HTA system should be designed to allow for stepwise adjustments and improvements over time. Success of the implementation should be consistently tracked and reported.

- Key Domains related to Challenges with Uncertainty:

A description of each of the domains and the listing of guiding questions are displayed in Table 2. Descriptive statistics (*n* = 23 responses) of the ranking of those domains are presented in Fig. 4.

**Table 2:** Identified key domains to address the challenge of uncertainty

Challenges with Uncertainty				
Key Domains	Early and inclusive collaboration	Agreement on feasibility of RCT and acceptance of uncertainty	Alignment on closing evidence gaps	Capacity gaps
Description	Collaboration should be enhanced especially at an early stage when designing the clinical development program. Continuous involvement of regulators, HTA bodies, clinicians, patients and industry is critical	Early exploration of feasibility of RCT should be agreed by all relevant stakeholders. If an RCT is considered not feasible alternative options of generation of comparative evidence should be explored and level of acceptable uncertainty should be agreed	Early agreement on comparative evidence gaps and how to generate data to appropriately address those gaps	Ensure sufficient capacities to allow for early and inclusive collaboration e.g., sufficient joint early advice opportunities
Guiding Questions	How can EU HTA take into account evolving clinical research and development paradigms in oncology to be a driver not a bottleneck for patient access to new medicines while at the same time not reducing methodological rigor? How can stakeholder involvement have meaningful impact?	Is an RCT possible in this context? What other options for generation of comparative data exist? How can additional types of evidence and methods help increase certainty? What level of certainty can be achieved with small sample sizes e.g., in childhood cancer or in ATMPs?	How to approach a gradually evolving evidence body with limited certainty at time of launch? What are suitable and achievable comparative evidence requirements for Real World Data and New Clinical Trial Designs?	What is the expected number of new oncology drug applications / year and the related joint scientific advice capabilities of EMA/ EU HTA? What can be done to increase the number of available joint scientific advice slots?

Identified core domains include: i) early and inclusive collaboration (mean 6.1; median 7; IQR 6–7), ii) agreement on feasibility of RCT and acceptance of uncertainty (mean 5.7; median 6; IQR 5–7), iii) alignment on closing of evidence gaps (mean 5.6; median 6; IQR 5–7), and iv) capacity gaps (mean 5.1; median 5; IQR 4–7).

Initially a fifth domain was suggested, covering: ‘value and costs of closing evidence gaps’. As content of this domain was closely related to ‘alignment on closing of evidence gaps’ the decision was made to merge the two domains. A key component regarding the first domain ‘early and inclusive collaboration’ is related to the increase of predictability throughout the process and to a potential improvement of the quality of assessments. However, as ‘early and inclusive collaboration’ extends beyond predictability the name of the domain was not changed. The second domain initially only focussed on an agreement on the feasibility of an RCT. However, in situations where an RCT is not feasible, e.g., in rare diseases or due to ethical considerations, acceptance of an alternative trial design, additional types of evidence and comparison methodologies that can help reduce risk and uncertainty might be required. Thus, the adjusted wording of the second domain includes a reference to acceptance of uncertainty. Evidence gaps are related to the uncertainty in evidence/ outcomes provided and therefore constitute another important domain in this area.

- Key Domains related to Challenges with Comparator Choice:

A description of each of the domains is displayed in Table 3. Descriptive statistics (*n* = 26 responses) of the ranking of those domains are presented in Fig. 5.

**Table 3:** Identified key domains to address the challenge of comparator choice

Challenges of Comparator Choice					
Key Domains	Criteria for the choice of comparator in an increasingly fragmented landscape	Reasonable number of comparators in PICOs	Shape Early Advice so that comparator fulfils both, regulatory and HTA needs	Acceptability of ITCs	Ensure broad stakeholder involvement in comparator selection
Description	Agreement across all involved stakeholders on the set of requirements for the appropriate comparative therapy within a clinical development program	The comparator should be the current standard of care. If multiple standards of care exist attempt should be made to limit the number of required comparisons	Agreement for early and inclusive advice covering both, regulatory and HTA needs	Agreement on applicability of ITC and methodological requirements for ITC	Ensure involvement and alignment across a wide stakeholder group incl. HTA, regulatory, clinical, patient, and industry stakeholders
Guiding Questions	How to manage an ever more diverging and fast-moving comparator landscape in targeted oncology? Is the current treatment standard on-label or off-label?	Is there a clear standard of care? How to manage different national Standards of Care and heterogeneous guideline recommendations? Is comparator selection based on best available alternative or other criteria? How should clinically interchangeable comparators be incorporated?	How to ensure that comparator choice fulfils both, regulatory and HTA requirements? What are alternative evidence sources and methods that can be used if required comparators differ, or a comparator cannot be ethically used with a trial?	Under what circumstances would an ITC be relevant? What are the contextual factors that can guide which ITC methods are most appropriate?	Who should be involved in the selection of appropriate comparative therapy? What are the rationales behind their choice?

Identified core domains include i) criteria for the choice of comparator in an increasingly fragmented treatment landscape (mean 6.0; median 6; IQR 5–7), ii) reasonable number of comparators in PICOs (mean 5.5; median 6; IQR 5–7), iii) shape early advice so that comparator fulfils both, regulatory and HTA needs (mean 5.5; median 6; IQR 5–7), iv) acceptability of Indirect Treatment Comparison (ITC) (mean 5.3; median 6; IQR 4–7), v) ensure broad stakeholder involvement in comparator selection (mean 5.3; median 6; IQR 4.75–6).

An additional domain named ‘how to manage comparator in basket trials’ was removed after discussion. The challenge of comparators in basket trials is nevertheless considered very relevant and conceptually covered by the reference to the increasingly fragmented treatment landscape in the first domain. This challenge arises in particular due to advances in developing targeted treatments – not exclusively but especially in the area of oncology, where biomarker-specific approaches to tumour treatment lead to small patient numbers with different tumour types that harbour the same genetic alteration and/or molecular pattern. The challenge with multiple PICOs is covered as a main process challenge. However, to keep the focus specifically on the challenge of multiple comparators within the different PICOs identified in the scoping phase, it was decided to retain the domain within the challenges with comparator choice, as well. Lack of sufficient early advice capacities was discussed within the context of ‘shaping early advice so that comparator fulfils both, regulatory and HTA needs’. The capacity issue is also included as a major process challenge. Finally, the acceptability of ITCs is related to the challenge of comparator choice as they might be required in the context of multiple comparators, but methodological requirements should be contextualized. However, the methodological requirements for ITCs are reaching beyond comparator choice only.

- Key Domains related to Challenges with Endpoint Selection:

A description of each of the domains is displayed in Table 4. Descriptive statistics (*n* = 26 responses) are presented in Fig. 6.

**Table 4:** Identified key domains to address the challenge of endpoint selection

Challenges with Endpoint Selection					
Key Domains	Approaching new endpoints	Patient preferences on endpoints	Position of HTA and other stakeholders	Long-term generation & secondary use of data	Endpoint challenges in RCTs
Description	Review of applicable endpoints at the commencement of each clinical development program and early agreement across all involved stakeholders if new endpoints are required in a particular setting	The view of patients should be taken into consideration for the determination of an endpoint as ‘patient-relevant’ in HTAs	Early collaboration should make the position of all involved stakeholders transparent and lead to resolution of differing views	Agreement on the need for the generation of long-term data and secondary use of data by all involved stakeholder groups	Review of and agreement on appropriate endpoints and feasibility of RCTs to generate comparative data on these endpoints between the involved stakeholders before commencement of the clinical program
Guiding Questions	Are new endpoints applicable to this disease area?	How to empower patients’ voice in the determination of patient-relevance of any specific endpoint?	Who should be involved in the definition of an endpoint? How can differing views on endpoint acceptability between stakeholders be resolved?	How can long-term data generation and secondary use of data be facilitated?	How can we ensure that endpoints match evolving treatment paradigms in an increasingly targeted oncology environment?

Identified core domains include i) approaching new endpoints (mean 5.7; median 6; IQR 5–7), ii) patient preferences on endpoints (mean 5.6; median 6; IQR 5–7), iii) position of HTA and other stakeholders (mean 5.4; median 6; IQR 4–7), iv) long-term generation and secondary use of data (mean 5.3; median 5; IQR 4–6), and v) endpoint challenges in RCTs (mean 4.8; median 5; IQR 4–6).

Regarding the first domain a discussion evolved whether to specify ‘definition and validation of new endpoints’. As validation did not seem appropriate in all situations relevant to HTA purposes the domain was finally named: ‘approaching new endpoints’, verbiage that includes the question whether new endpoints will be accepted by HTA bodies in a given clinical context. Although patients are also included as ‘other stakeholders’ in the third domain the determination of patient preferences with regards to endpoints was deemed particularly important and kept as a separate domain. Discussion of who should contribute to the determination of endpoints that are relevant to patients in a specific disease setting is the intent of the third domain, which had initially focussed on the role of HTA in endpoint selection only. During the discussion it became clear that early collaboration across all relevant stakeholders should be aimed for. Long-term generation of endpoint data and secondary use of data e.g., from disease registries, were originally considered two separate domains but merged into one domain as both concepts are closely related. The domain ‘endpoint challenges in RCTs’ covers the feasibility and challenges of generating comparative data on an acceptable endpoint which is relevant to patients, used in clinical practice and mature enough in the readout, in a specific disease context in an RCT. It is closely related to the concept of challenges with uncertainty, however, it received the lowest scores compared to the other domains. While the perception was that the first domain (‘approaching new endpoints’) covers some key aspects of endpoint challenges in RCTs it was still decided to retain this domain as a separate item.

## Discussion

The aim of a joint European HTA assessment is stated early on in §3 of the regulation: *‘HTA is able to contribute to the promotion of innovation, which offers best outcomes for patients and society as a whole, and is an important tool for ensuring proper application and use of health technologies’* [1]. As has been repeatedly shown the challenge with the current—pre-regulation—status quo is a high level of heterogeneity of the various national and sub-national European HTA assessments resulting in different access to innovation across the EU member states and lack of predictability and a multiplicity of national HTA submissions on the side of the HTDs [6, 7]. As has been shown e.g., within the ‘SIOPE Access to Medicines Project’, ample variability in HTA decision making across Europe has an impact on availability of anticancer medicines for highly vulnerable patient groups [8]. The new EU HTA regulation is a unique opportunity to consolidate the various national HTA approaches and shape processes and methods to strengthen the European Health Union and to ensure that *‘the development of health technologies is a key driver of economic growth and innovation in the Union and is key to achieving the high level of health protection that health policies need to ensure for the benefit of all’* as stated in §1 of the regulation [1].

The presented research agenda is aimed at highlighting key challenges that warrant further research and resolution to fulfil the intentions of the regulation. While challenges were grouped into process challenges and methodological challenges (uncertainty/ comparator/ endpoints), setting up the right processes and resolving key issues in the respective domains were considered priority as they are also shaping the subsequent approach to the methodological requirements:

- The challenge of multiple PICOs was considered a major hurdle for a harmonization of EU HTA efforts. Currently, the EU regulation is ambiguous as it suggests both to ‘*harmonize transparent HTA criteria to assess the added therapeutic value… compared with the best available alternative*’ [1, 9] and that ‘*in particular member states should be able to perform complementary clinical analysis relating… to…comparators… other than those included in the joint clinical assessment report…*’ [1, 15]. Where the Original Proposal for an EU HTA Regulation stated *‘Ensure the use of joint outputs in Member States’* as an Operational Objective [9] this wording is not included in the final EU HTA regulation [1]. To what extent ITCs can overcome the challenges resulting from multiple PICOs was discussed in the working group comparator choice. Therefore, ‘Acceptability of ITCs’ was selected as another domain requiring further research and resolution. Previous efforts from the EUnetHTA group to establish a European PICO [10] had aimed to implement a transparent PICO survey. However, divergence in the comparator choice remains [11], in particular due to differences in national health systems and varying levels of availability of medical treatments. This divergence will continue to make it very difficult for HTDs to shape a common clinical development program that is addressing all stakeholder needs. Therefore, it has to be considered a key objective e.g., for the developing Coordination Group and its sub-groups to not just to coordinate the assemblage of different PICOs but instead to work towards a convergence of PICO requirements in order to increase feasibility and predictability of assessment, as well as deliver a high quality report within the timeframe outlined in the regulation. Without doubt this process will cause conflicts, both between various European stakeholders (e.g., HTA bodies, regulators, medical societies, patient associations and industry) and between European and national HTA bodies. However, the success of the common EU assessment will largely depend on the capability to orchestrate this conflict in the spirit of the EU HTA Regulation. The domain ‘conflicts and alignment’ may therefore almost be considered a key performance indicator for the evolving EU HTA body.
- Time and capacity challenges were considered another major limitation for a successful implementation of the new regulation. As indicated in the EUnetHTA 21 stakeholder call on March 25^th^, 2022, it is planned to conduct just one to two JCAs in 2023 and a very limited number of JSCs [3]. Stepwise upscaling, mutual learning and milestone tracking are important measures when implementing a new system but were not included in the scope and resources as set out in the tender specifications for the EUnetHTA 21 project by the European Commission [2]. As development of innovative oncology medicines and ATMPs is frequently based on very targeted approach with small patient numbers or where randomization is not possible, sufficient advice capacities to e.g., ensure early and inclusive collaboration across all stakeholder groups to cover methodological uncertainty challenges are critical.
- Comprehensive stakeholder involvement throughout the whole process is another key topic shaping the prospects of the EU HTA regulation [12]. The stepwise establishment of a European HTA system will result in new requirements and interfaces not just for HTDs but also for patient associations, medical societies, regulators, and industry. Indeed, HTA bodies have already defined patient-relevant endpoints used for relative effectiveness assessment in Europe [13]. However, it appears important to include all relevant stakeholders in the definition of trial endpoints that are relevant to patients in a specific disease setting [14–16], as well as determination of the appropriate comparator [17]. The question remains how to approach the challenge of what level of uncertainty may be considered acceptable in disease setting e.g., what might constitute appropriate criteria, who would be involved in making a decision, etc. Such societal questions are reaching far beyond technical discussions between HTDs and HTA bodies and require involvement of a wide spectrum of stakeholders. The recently published joint EMA/ EUnetHTA work plan may be considered an important first step in the direction of comprehensive stakeholder involvement [18–20]. Collaboration with medical societies, patient representatives and industry will require similar systematic work plans to ensure societal convergence on these key value considerations.

The initial implementation phase of the EU HTA regulation is focusing on Oncology and ATMPs. This scope matches the intention of ‘Europe’s Beating Cancer plan’ [21] and reflects the high level of unmet medical need in these fields. However, research in oncology has advanced to ever more targeted interventions in ever smaller populations. Consequently, innovative oncology medicines will also require innovative methodologies to determine the additional benefit over the current standard of care in such small populations [8, 22]. Furthermore, certain rare conditions, as well as technologies such as ATMPs, might not allow for the conduct of a RCT. Agreement on alternative options to collect comparative data [23] as well as discussions on the acceptable level of uncertainty in evidence generation in a certain disease context might therefore be required [24, 25].

## Conclusion

The implementation of a joint European HTA assessment is a unique opportunity for a strong European Health Union. We identified 19 domains related to the four key areas processes, uncertainty, comparator choice and endpoint selection that urgently need to be addressed for this regulation to become a success. Considering many overlapping issues and challenges, an integrated and coordinated strategy including all relevant stakeholders is needed. A continuous tracking of the regulations’ implementation milestones will be required to ensure that this HTA assessment is *able to contribute to the promotion of innovation, which offers best outcomes for patients and society as a whole*.

## Data Availability

The datasets used and/or analysed during the current study are available from the corresponding author on reasonable request.

## Abbreviations

ATMP: Advanced Therapy Medicinal Product
EAA: European Access Academy
HTA: Health Technology Assessment
HTD: Health Technology Developer
IQR: Interquartile Range
ITC: Indirect Treatment Comparison
JCA: Joint Clinical Assessment
JSC: Joint Scientific Consultation
PICO: Patient/ Intervention/ Comparator/ Outcomes
RCT: Randomized Controlled Trial
SIOPE: European Society for Paediatric Oncology
ZIN: Zorginstitut, Netherlands
